# Squill oil for decreasing dyspareunia and increasing sexual satisfaction in menopausal women: A triple-blind randomized controlled trial

**DOI:** 10.22038/AJP.2021.17777

**Published:** 2021

**Authors:** Farzane Karimi, Raheleh Babazadeh, Abbas Zojaji, Samaneh Jouya

**Affiliations:** 1 *Department of Midwifery, Birjand branch, Islamic Azad University, Birjand, Iran *; 2 *Nursing and Midwifery Care Research Center, Mashhad University of Medical Sciences, Mashhad, Iran*; 3 * Department of Midwifery, School of Nursing and Midwifery, Mashhad University of Medical Sciences, Mashhad, Iran*; 4 *Department of Medicine, Mashhad branch, Islamic Azad University, Mashhad, Iran*; 5 *Department of Midwifery, School of Nursing and Midwifery, Mashhad University of Medical Sciences, Mashhad, Iran*

**Keywords:** Menopause, Post menopause, Sexual, Phytoestrogen, Herbal

## Abstract

**Objective:**

The present study aimed to investigate the effect of squill oil on dyspareunia and sexual satisfaction in menopausal women.

**Materials and Methods:**

The present triple-blind randomized two-group controlled trial was conducted on 60 menopausal women (n=30 in placebo group and n=30 in squill oil group) in Mashhad in northeast of Iran in 2019. The Sabbatsberg Sexual Self-Rating Scale and Marinoff dyspareunia scale were the main tools used in this study. The participants were randomly assigned to two groups namely, squill oil group and placebo group and they received the treatments for 4 weeks. Questionnaires were completed before and 4 weeks after the intervention in both groups. Data analysis was performed using SPSS 24 by Mann-Whitney, t-test, and repeated measures ANOVA with a significance level of less than 0.05.

**Results:**

The average age in the squill oil group and placebo group were 53.5±2.2 and 51.7±4.5 years, respectively. There was a significant difference (p<0.001) between the two groups in terms of dyspareunia score before (1±3.6 vs 1±3.5) and after intervention (0.7±0.1 vs 1.2±1.2) in two groups.

Results of independent t-test indicated that there was a significant difference (p<0.001) between the two groups in terms of sexual satisfaction before (23.4±5.7 vs 23.1±2.8) and after intervention (36.5±5.6 vs 24.8±2.5) in two groups.

**Conclusion:**

Using squill oil can cause a reduction in painful sexual intercourse and an increase in sexual satisfaction in postmenopausal women.

## Introduction

Menopause means amenorrhea for 12 months and termination of women's fertility (Minkin, 2019 ▶). Almost one-third of the female populations in the US are in the post-menopausal period and at risk of menopausal symptoms. The mean age of menopause is 51.5 years in the United States (Truong et al., 2019 ▶). The average age of menopause is 48.2 years in Iranian women (Nahidi et al., 2010 ▶), and their life expectancy is 74.6 years (Golzareh et al., 2017 ▶). The population of women aged 50 and over, that seems to be menopausal, is 9.31 million, comprising 36.8% of total female. The proportion of women aged 50 to 64 years is 21.5% and 65 years and over is 15.2% (Kim et al., 2015 ▶)

The prevalence of sexual disorders in postmenopausal women is four times higher than women during the reproductive years (Malakouti et al., 2016 ▶). In fact, dyspareunia or pain during intercourse is one of the most common problems in postmenopausal women and can lead to discomfort, lower sexual function and pleasure, communication problems, and lower quality of life (Kao et al., 2008 ▶). Dyspareunia is usually due to a decrease in the secretion of ovarian hormones. Due to the aging of the vaginal tissue and decrease in the level of estrogen produced during menopause, especially estradiol (E2), atrophic changes may be seen in the external genitalia, anus, and vagina, which may include itching and dryness of the vagina and dyspareunia.

On the other hand, sexual satisfaction is an important factor in the marital satisfaction (Malakouti et al., 2016 ▶) that decreases under the influence of dyspareunia (Binik, 2010 ▶). Vaginal lubricants can be a treatment option for postmenopausal women who need a vaginal slippage to prevent discomfort and pain during intercourse (Kingsberg et al., 2009 ▶). 

Acceptance of synthetic estrogen therapy is low mainly due to concerns about its complications (cardiovascular events, breast cancer, and endometrial hyperplasia) (Poluzzi et al., 2014 ▶). According to the World Health Organization, 80% of people use herbs for their health problems (Bensky et al., 2004 ▶). Among alternative and complementary therapies, herbal therapy and use of the estrogenic plant (estrogen-containing compounds) have a special place in the treatment of certain problems caused by menopause (Mirghafourvand et al., 2015 ▶). Phytoestrogenic compounds are weaker than conventional estrogens and have been introduced as safer and more effective alternatives (Jefferson et al., 2007 ▶). 

Squill is a herb that has attracted the attention of traditional medicine and researchers due to its numerous properties. Squill bulb is pear-shaped and has a diameter of 3-15 cm. The plant was formerly considered a member of the Liliaceae family (Biruni, 1990 ▶) It is distributed all over the world, including rocky beaches, especially in the Mediterranean regions, southern Europe, North Africa, and Southwest Asia, and Iran in the Persian Gulf beaches and oak forests of the *Zagros* Mountains (Biruni, 1990 ▶; Bozorgi et al., 2017 ▶). Its scientific name is *Drimia maritima* and it is also known as Sea Onion. Flavonoids have been identified in squill extract (Gutiérrez et al., 2008 ▶). Squill is available in two colors: white and red (Biruni, 1990 ▶). 

A type of squill is more suitable for medication and is pearly white and bright. It is applied in traditional medicine because of its warm and dry nature. The main applications of Drimia species are in car-diovascular disorders, respiratory ailments, bone and joints complications. It also has proper anti-oxidant and anti-inflammatory effects and has detoxification ability (Bozorgi et al., 2017 ▶). 

Squill oil, as an oil with warm nature, dilates the blood vessels, increases neural and peripheral blood flow, and increases sexual desire and libido (Aghili. MH, 2008 ▶). The active derivatives of this oil are phytosterols and a flavonoid with phytoestrogenic properties called quercetin. Along with the flavonoids in the plant, Quercetin produces phytoestrogenic effects that enhance sexual desire, arousal, and lubrication. Due to similar structures of phytoestrogens to estradiol, they can imitate estradiol or have agonistic properties on its receptors (Karabin et al., 2015 ▶). 

The present study aimed to investigate the effect of squill oil on dyspareunia and sexual satisfaction in postmenopausal women.

## Materials and Methods

The present triple-blind two-group randomized controlled trial was conducted on 60 postmenopausal women in Imam Reza Hospital of Mashhad University of Medical Sciences; Subjects were randomly taken from those who were referred to Imam Reza Hospital as a referral center. Inclusion criteria were as follows: Age between 45-65 years; literacy; The last menstrual period occurred a year ago; *Follicle-stimulating hormone* (FSH) of greater than 40 mIU/mL ; complaining of dyspareunia (earning at least one score based on the Marinoff dyspareunia scale); women with sex and a spouse; sex at least once or twice a week; symptoms of vaginal atrophy (vaginal dryness and paleness, and painful intercourse); no hormone therapy, or sex hormone use during 8 weeks prior to the study; no smoking and alcohol use; no abnormal uterine or vaginal bleeding; and no over-use of other phytoestrogenic drugs (soy, red clover, fenugreek, *Vitex agnus-castus*, or fennel) over the past month; and no breast disease with unknown cause. Exclusion criteria: vaginal infection (bacterial vaginosis, trichomoniasis) and any major disease of the genital tract. Pelvic inflammatory disease (PID), uterine prolapse, cystocele, rectocele that were approved by the first researcher during the study; not taking the drug completely by patient; or itching and skin allergy symptoms due to the drug consumption.

The instruments included demographic and marital questionnaire, Sabbatsberg Sexual Self-Rating Scale, and Marinoff dyspareunia scale. 

The demographic and marital questionnaire consisted of 20 questions. To assess the content validity, the questionnaire was distributed to 10 faculty members of Mashhad Faculty of Nursing and Midwifery and was applied after reviewing their views.

The Sabbatsberg Sexual Self-Rating Scale of twelve 5-choice questions including 4 questions about satisfaction and pleasure; 6 questions about the importance, frequency and interest in sex; and 2 about the ability to reach orgasm; and its total score indicates the rate of sexual satisfaction. Content and scientific validity of the English version were assessed by Garratt et al. with a Cronbach 's alpha coefficient of 0.95 (Garratt et al., 1995 ▶). The validity and reliability of translated tool were confirmed in by Taavoni et al. in Iran (Taavoni et al., 2005 ▶). Marinoff dyspareunia scale included 4 choices with a score of 0-3 (score 0 = no pain during intercourse, score 1=pain during intercourse that does not prevent the intercourse, score 2=pain during intercourse that interrupts intercourse, and score 3=pain that prevents the intercourse). the present study (Marinoff et al., 1993 ▶). Reliability of the scale was obtained with a Cronbach's alpha of 0.94.

Sample size was calculated based on a study by Abbasi et al. (Abbasi Pirouz et al., 2018 ▶) and it was estimated to be 26 per group in the comparison of two independent populations with a 90% power and 95% confidence coefficient. The sample size was considered 30 per group with a probability of 20% sample loss.

Samples were selected according to the inclusion criteria by convenience sampling from women who visited Imam Reza Women Clinic. After explaining the research purpose, obtaining their informed consent and completing demographic form, Sabbatsberg Sexual Self-Rating Scale, and Marinoff dyspareunia scale, women were randomly assigned to the placebo or squill oil groups. The randomization was arranged by a member of the research team (second author), 30 letters A and 30 letters B with the help of https://random-ize.com/. squill oil tubes (squill oil group) and olive oil (placebo) were numbered in closed, non- transparent envelopes, and were packaged similarly with labels A and B of which only a pharmacy consultant was aware. Also, a training pamphlet on the way of medication and genitalia and clitoris anatomy in simple language was given, and each person was given an envelope contain tube of oil for consumption for 4 weeks according to the sequence established. Envelopes were prepared and given by a person non-involved in data collection or analysis. Therefore, the patients, prescriber, data collector, and data analyst were not aware of the type of intervention. 

The tubes of the oils were similar in appearance and were prepared by the pharmacist (first wash the squill bulb with cold water until its dust was thoroughly cleaned. About 900 g of the squill was mixed with 3 kg of olive oil, and was then heated for 10 hr at 50°C and finally its oil was filtered) (Aghili, 2008 ▶).

The patients in the squill oil group and placebo groups used 3 ml of oil (equivalent to 1 teaspoon) gently on the clitoris and vaginal opening for 2 to 3 times per week, 5 min before the intercourse for 4 weeks. Thereafter, the number of oil use and its side effects were followed up by making phone calls each week (for 4 weeks). After 4 weeks, the participants were contacted to visit and re-complete the Sabbatsberg Sexual Self-Rating Scale and Marinoff dyspareunia scale. At the end of the study and in case of non-recovery or complaint about the dyspareunia, patients were referred to a specialist. Dyspareunia was the primary outcome and the sexual satisfaction was the secondary outcome that was measured using relevant questionnaires at baseline and 4 weeks after the intervention. Data analysis was performed using SPSS 24 and the repeated measures ANOVA, Mann-Whitney test, and t-test. The present study was conducted after registering in the Iranian Registry of Clinical Trials (https://www.irct.ir with registration No. IRCT20170430033718N4 and obtaining written consent from subjects.

## Results

There was no statistically significant difference between the two groups in terms of mean age, spouse age and marriage age (Table 1). Mann-Whitney test indicated no significant difference between the two groups in terms of dyspareunia before the intervention (p=0.433). Mann-Whitney test indicated that the dyspareunia was significantly lower in the squill oil group than the placebo group after the intervention (p=0.017). Results of Kruskal-Wallis test indicated that changes in dyspareunia significantly decreased after intervention compared to the pre-intervention in both squill oil group (p<0.001) and placebo group (p=0.002) groups (Table 2).

The repeated measures analysis of variance (ANOVA) was used to compare sexual satisfaction scores before and after the intervention. The time (p<0.001), intervention (p<0.001) and the interaction between time and intervention (p<0.001) had significant effects on the scores of Sabbatsberg Sexual Self-Rating Scale and its subgroups (Table 3). The results of independent t-test indicated that the score of Sabbatsberg Sexual Self-Rating and its subgroups in squill oil group at the post-intervention stage was significantly higher than the placebo group (p<0.001) (Table 3).

**Table1:** Demographic characteristics of the participants^*^

**p value**	**Squill oil ** **(n=** **30** **) Placebo (n=30)**		**Characteristic**
0.103=p	4.5±51.7	53.5±2.2	Age(y)
0.499	5.5±57.4	5.5±57.1	Husband age(y)
0.902	1.1±2.5	1.1±3.5	Pregnancy(n)
0.544			**Education level **
	17 (56.67)	12 (40.0)	Elementary
	3 (10.0)	5 (16.67)	middle school
	8 (26.67)	1 1(36.66)	high school
	2 (6.66)	2 (6.67)	University education
0.113			**Education level of spouse**
	8 (26.66)	7 (23.33)	Elementary
	9 (30.0)	8 (26.66)	middle school
	4 (13.33)	7(23.33)	high school
	9 (30.0)	8 (26.66)	University education
0.407			**Woman's job**
	29 (29.0)	26 (26.0)	Household
	1 (1.0)	4 (4.0)	Employed
0.265			**Husband's job**
	17 (50.0)	17 (65.0)	Self-employed
	7 (7.5)	6 (10.0)	Worker
	6 (42.5)	7 (25.0)	Retired
0.340			**Socio-economic class**
	8 (26.67) 15 (50.0)	5 (16.66) 14 (46.67)	Low Moderate
	7 (23.33)	11 (36.66)	High
0.157			**Sport activity**
	11 (36.67)	16 (53.33)	yes
	19 (63.33)	14 (46.67)	No
0.251			**Separate room for sex**
	26 (86.67)	28 (93.33)	Yes
	4(13.33)	2 (6.67)	No
0.754			**Satisfaction with marriage**
	4 (13.33)	2 (6.67)	Very satisfying
	9 (13.33)	10 (33.33)	Low satisfaction
	7 (23.33)	6 (20.0)	Very low satisfaction
	10 (33.33)	12 (40.0)	No satisfaction
0.481	1.8±2.5	3.4±4.0	**Last menstrual period** (y)
1.000	2.5±4.1	2.8±4.3	**Sex per month**
1.000	27 (90.0)	28 (93.33)	**Vaginal delivery** yes
	3 (10.0)	2 (6.67)	No

*Data is presented as mean±SD and number (percent). Statistical analyses were done using Independent T-test.

**Table 2:** Comparison of dyspareunia according to Marinoff scales between Squill oil and placebo group

**Variable**	**Group**		**Total** ** N (%)**	**p** ^ (4)^ **(between group)**
	**Squill oil**	**Placebo ** **N (%)**		
	**N (%)**			
Baseline				0.433
no pain with intercourse	0 (0.0)	0 (0.0)	0 (0.0)	
Scale 1^(1)^	13 (43.3)	17 (56.7)	30 (50.0)	
Scale 2^(2)^	15 (50.0)	10 (33.3)	25 (41.7)	
Scale 3^(3)^	2 (6.7)	3 (10.0)	5 (8.3)	
After				0.017
no pain with intercourse	10 (33.3)	6 (20.0)	16 (26.7)	
Scale 1^(1)^	18 (60.0)	13 (43.3)	31 (51.7)	
Scale 2^(2)^	2 (6.7)	10 (33.3)	12 (20.0)	
Scale 3^(3)^	0 (0.0)	1 (3.3)	1 (1.7)	
p^ (5)^ (within group)	<0.001	0.002	<0.001	

(1) The presence of pain with intercourse that does not prevent the completion, (2) The presence of pain with intercourse requiring interruption or discontinuance, (3) The presence of pain with intercourse preventing any intercourse (4) Mann–Whitney U test, (5) Wilcoxon signed-rank test

**Table 3:** The Mean±SD of the variables before and after the intervention in the two groups

**Variables**	** Group**				**Total**		**p** ^ (1)^	**p** ^ (2)^		
	**Squill oil**		**Placebo**							
	**n**	**Mean±SD**	**n**	**Mean±SD**	**n**	**Mean±SD**		**Time**	**Treatment**	**Time×** **Treatment**
Sabbatsberg (interest)								<.001	<.001	<.001
Before	30	12.4±3.2	30	11.5±1.8	60	11.9±2.6	.204			
After	30	18.3±3.7	30	12.3±1.6	60	15.3±4.1	<.001			
After – Before	30	6.0±2.7	30	0.8±2.2	60	3.4±3.6	<.001			
Sabbatsberg (satisfaction)								<.001	<.001	<.001
Before	30	7.7±2.2	30	7.9±1.5	60	7.8±1.9	.631			
After	30	12.0±2.4	30	8.4±1.4	60	10.2±2.7	<.001			
After – Before	30	4.4±1.6	30	0.5±1.7	60	2.4±2.6	<.001			
Sabbatsberg (orgasm)								<.001	<.001	<.001
Before	30	3.4±1.2	30	3.7±1.0	60	3.6±1.1	.286			
After	30	6.1±0.9	30	4.1±1.0	60	5.1±1.4	<.001			
After – Before	30	2.7±1.1	30	0.4±0.8	60	1.6±1.5	<.001			
Sabbatsberg (Total)								<.001	<.001	<.001
Before	30	23.4±5.7	30	23.1±2.8	60	23.3±4.5	.777			
After	30	36.5±5.6	30	24.8±2.5	60	30.7±7.3	<.001			
After – Before	30	13.1±3.3	30	1.7±3.4	60	7.4±6.6	<.001			

1) Intergroup test (Independent t-Test) 2) Repeated measures analysis of variance

## Discussion

In the present study, squill oil and placebo (olive oil) were used on postmenopausal women for 4 weeks and it was found that dyspareunia was significantly lower in the squill oil group than the placebo group after using the squill oil. The squill oil also increased sexual satisfaction in postmenopausal women in the squill oil group compared to the placebo group. Various studies have indicated that women's age, education level 

employment status, economic status, number of children, and weight and lifestyles might affect their physical symptoms in postmenopausal (Golzareh et al., 2017 ▶). In this study, two groups that were homogeneous in terms of individual characteristics were considered.

To our knowledge, there was no study on the effect of consumption of squill oil in postmenopausal women. In the present study, topical use of squill oil on the clitoris decreased dyspareunia and increased sexual satisfaction in postmenopausal women by increasing the blood supply and through phytoestrogenic effects. In a study by Abbasi et al., the use of squill oil in women at childbearing ages increased the sexual function score and its subgroups (desire, arousal, lubrication, pain in intercourse, and orgasm) (Abbasi Pirouz et al., 2018 ▶) and the result was consistent with the present study. 

Fennel vaginal cream in a study by Najar et al. and fenugreek vaginal cream in a study by Mazalzadeh et al. (2015) ▶ had phytoestrogenic properties that decreased dyspareunia and increased sexual satisfaction in postmenopausal women (Mazalzadeh et al., 2018 ▶; Najar et al., 2015 ▶); their results were consistent with results of the present study. In a clinical trial study by Taavoni et al. (2016) ▶, the women's sexual desire and orgasm improved 4 weeks after using Afrodit. (Herbal Drop contain: Bindii, Cinnamon, Saffron, Ginger). In another study by Taavoni et al. (2013) ▶, *Ginkgo biloba* extract oil, which has phytoestrogens, improved females libido (Taavoni et al., 2016 ▶). In a study by Azimipour et al. (2017) ▶, the use of Cimifugol tablet (dry root of *Cimicifuga racemosa*), which has phytoestrogenic properties, was effective in improving the genitourinary symptoms (sexual dysfunction, vaginal dryness, and urinary symptoms) in postmenopausal women.

Their results were consistent with the present study (Azimipour et al., 2017 ▶). In a study by Yousefzadeh et al. (2017) ▶, the use of date palm pollen capsule with its phytoestrogenic properties had no effect on sexual satisfaction. The drug was used as an oral capsule in Yousefzadeh et al. study, but it was topically used in the present study (Yosefzadeh et al., 2017 ▶).

In a study by Pamenari et al. (2015) ▶ on 76 females of 19-58 years old, Femore gel (topical L-arginine) and placebo gel equally improved women's sexual function (Pamenari A et al., 2015 ▶). Femore gel is an expensive drug in Iran, but it had no significant difference with effect of placebo according to the research above, while the frequent use of topical squill oil could decrease dyspareunia and increase satisfaction in postmenopausal women by creating the lubrication through increasing local blood flow compared to placebo (olive oil). According to data of a research by Kingsberg et al., most women experienced discomfort during the use of vaginal cream and found it dirty (Kingsberg et al., 2017 ▶) the use of herbal oils with less topical external use is less likely to be acceptable for women. 

The innovation of the present study was the use of a herbal topical oil to reduce dyspareunia and increase sexual satisfaction in postmenopausal women. Strengths of this study include the use of a validated outcome measure. One of the limitations of this study was the lack of control over all factors affecting sexual satisfaction, including mental states in the subjects, for which the placebo group could not help. It is suggested to conduct a research on the effect of this herb on the treatment of inflammatory vaginitis in postmenopausal women. In final, squill oil could be considered an effective herb with phytoestrogenic properties to reduce dyspareunia and increase sexual satisfaction in postmenopausal women.
